# Primate protein-ligand interfaces exhibit significant conservation and unveil human-specific evolutionary drivers

**DOI:** 10.1371/journal.pcbi.1010966

**Published:** 2023-03-23

**Authors:** Sean B. King, Mona Singh

**Affiliations:** 1 Department of Molecular Biology, Princeton University, Princeton, New Jersey, United States of America; 2 Lewis-Sigler Institute for Integrative Genomics, Princeton University, Princeton, New Jersey, United States of America; 3 Department of Computer Science, Princeton University, Princeton, New Jersey, United States of America; National Center for Biotechnology Information (NCBI), UNITED STATES

## Abstract

Despite the vast phenotypic differences observed across primates, their protein products are largely similar to each other at the sequence level. We hypothesized that, since proteins accomplish all their functions via interactions with other molecules, alterations in the sites that participate in these interactions may be of critical importance. To uncover the extent to which these sites evolve across primates, we built a structurally-derived dataset of ~4,200 one-to-one orthologous sequence groups across 18 primate species, consisting of ~68,000 ligand-binding sites that interact with DNA, RNA, small molecules, ions, or peptides. Using this dataset, we identify functionally important patterns of conservation and variation within the amino acid residues that facilitate protein-ligand interactions across the primate phylogeny. We uncover that interaction sites are significantly more conserved than other sites, and that sites binding DNA and RNA further exhibit the lowest levels of variation. We also show that the subset of ligand-binding sites that do vary are enriched in components of gene regulatory pathways and uncover several instances of human-specific ligand-binding site changes within transcription factors. Altogether, our results suggest that ligand-binding sites have experienced selective pressure in primates and propose that variation in these sites may have an outsized effect on phenotypic variation in primates through pleiotropic effects on gene regulation.

## Author summary

Humans are very similar to other primates with respect to their genome sequences, yet exhibit striking phenotypic differences. We hypothesized that changes in a small number of important positions within the protein coding region of the genome may contribute to these phenotypic differences. We focused our study on evaluating the evolution of sites within proteins that interact with ligands, as variation at these sites has outsized potential for broader downstream effects. To uncover the extent to which ligand-binding sites have evolved across primates, we built a large database of sites within proteins that interact with DNA, RNA, small molecules, ions, or peptides. Using this dataset, we have shown that ligand-binding sites evolve more slowly than other protein residues, and that this is particularly pronounced for DNA and RNA binding sites. We have also shown that those ligand-binding sites that do vary are enriched in gene regulatory pathways, and that amino acids that are divergent in humans, but fixed among other primates, are found in several transcription factors. Our work aligns with long-standing evidence suggesting that regulatory networks influence phenotypic differences between primates, while uncovering novel contributions of ligand-binding sites to the evolution of these networks.

## Introduction

There is vast phenotypic variability among species across the tree of life. While some genes are highly conserved across living organisms (e.g., ribosomal genes [1]), most of the genome is capable of variation, and some of this variation drives the phenotypic differences observed across organisms. Within primates, we observe divergent lifecycles, body size and development, sensory perception, and social behavior [2,3]. Even between humans and other apes, there are substantial differences ranging across craniofacial features, cognition, immune system function, and even karyotype [4,5,6,7,8]. Further, there are notable species-specific differences in gene expression patterns across primates, especially in the brain [9,10].

Despite considerable differences in phenotypes among primate species, there is surprisingly little genetic variation between these species. For instance, the total genomic divergence between humans and their closest primate relatives, chimpanzees, is less than 4% [11,12,13], with orthologous proteins typically differing in only a couple of amino acids [14]. Because of this, it has been proposed that the primary driver of phenotypic variability across primates is due not to changes within protein sequences, but instead due to changes that affect how proteins are regulated [15,16,17,18]. While cis-regulatory alterations indeed have proven critical to a variety of morphological, developmental, and physiological changes across organisms [4,19,20,21,22], it is also well established that even small amounts of variation in key amino acid positions within proteins can potentially have large effects (e.g., [23,24,25,26,27]). This is evident, for example, by the small number of alterations that can transform healthy cells into cancerous cells, often by altering the interactions that proteins make with their ligands [25,28,29,30,31,32,33]. Thus, it is also quite possible that some of the phenotypic variation observed across primates is due to critical changes within protein sequences.

Here, we sought to uncover functionally important amino acid variation across primate protein sequences. Since many evolutionary changes within protein sequences are neutral, we focus on a class of alterations that most likely have significant biological effects. Specifically, since proteins accomplish nearly all of their functions via interactions with other molecules, variation in the sites within a protein that participate in its interactions can greatly impact its function. We chose to characterize changes in interaction sites in one-to-one orthologs, as these orthologs are under selection to maintain their ancestral functions; duplicates, on the other hand, can harbor functionally important amino acid variation within an organism to accomplish distinct functions, making their analysis more difficult.

Previous work to understand protein interaction variation across species has focused on the rate of gains and losses of interactions across species, and has been estimated using large-scale maps of protein-protein, protein-DNA, and to a lesser extent protein-RNA interactions that have been determined across the evolutionary spectrum [34,35]. While the presence of interactions between two proteins in one organism is often used to predict interactions between their homologs in other organisms (interologs) [36,37], it has also been proposed that there is considerable gain and loss of protein-protein interactions over evolutionary time [38]. The rate with which proteins gain or lose interactions is lower than the rate of sequence evolution [39], though it has been observed that protein sequence divergence is correlated with protein interaction divergence [40]. Previous studies of the evolution of regulatory interactions have revealed that they tend to be more plastic than protein-protein interactions [41], with orthologous transcription factors binding different targets across species [42,43]. Most work characterizing the variation of regulatory interactions has focused on the evolution of non-coding regions of the genome, along with duplication or loss events of transcription factor genes, and changes in protein-protein interactions [15,16,17,44,45,46,47,48,49]. Within primates, gene regulatory pathways in early neural crest development have been directly linked to the evolution of the craniofacial features unique to humans [4]. Further, it has been shown in *Drosophila* that their neural complexity can be partially attributed to a significant protein family expansion that encompasses cell-adhesion molecules, surface receptors, and their ligands, which has provided considerable insight into the rise of the metazoan nervous system [50].

While these previous studies have analyzed the variation of protein interactions and the networks in which they participate, knowledge of the individual amino acids participating in these interactions is generally more limited as granular structural information is necessary. Joint evolutionary and structural analyses of protein-protein interactions have revealed that residues involved in protein-protein interaction interfaces are more conserved than surface residues, suggesting that these residues are under purifying selection [51,52]. Further, sites involved in obligate interactions are somewhat more conserved than those involved in transient interactions [53]. Most DNA-binding sites within orthologous transcription factors are thought to be conserved [54,55], as changes within them would result in pleiotropic effects. However, the evolution of *Drosophila* transcription factors has provided evidence that DNA-binding sites in single-copy Cys2His2 zinc finger transcription factor proteins can vary considerably across these flies [56]. Strong phenotypic consequences have also been observed for variation within *Drosophila* developmental regulators, where a single amino acid change within an interaction site within a homeodomain protein can significantly alter embryonic body patterning [57].

**Fig 1:**
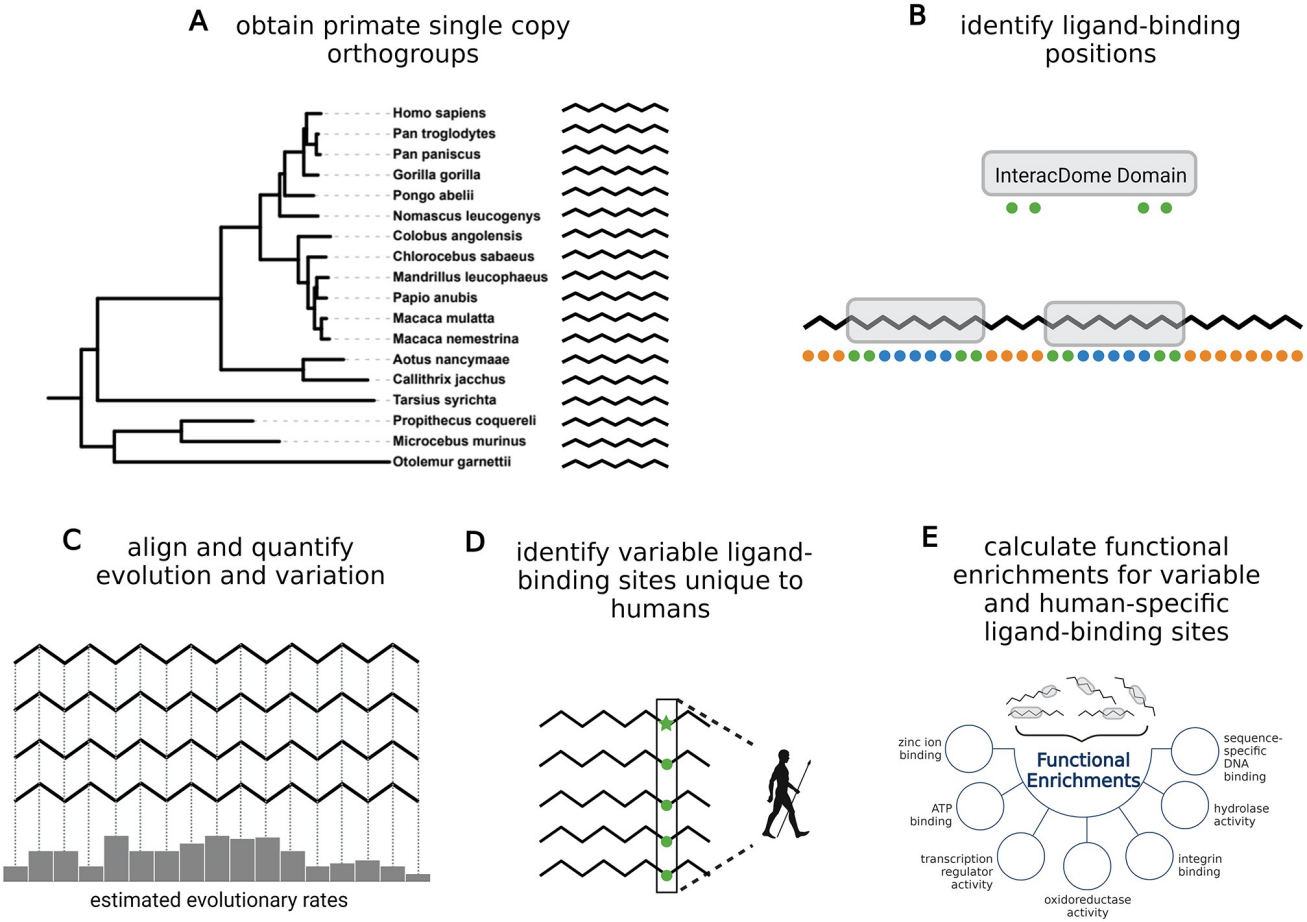
Overview of pipeline for characterizing ligand-binding site evolution in primates. **(A)** Single-copy orthogroups from 18 primate species were obtained from OrthoDB [61]. The phylogenetic tree of the 18 primate species considered is shown, and includes six apes, eight monkeys, and four prosimians; the tree is extracted from a molecular phylogeny of 186 extant primates [64] and visualized using the iTOL platform [65]. **(B)** For each orthogroup, domain-level site-wise ligand-binding data from the InteracDome [58] was transferred from domain space to the primary sequence space across sequences within each orthogroup. Green dots under the domain specify the ligand-binding positions within the domain. Grey boxes show instances of InteracDome domains within a sequence. Within that sequence, green depicts residues annotated as ‘ligand-binding sites,’ blue depicts residues within domains that are annotated as not ligand-binding sites and are annotated as ‘other sites within domains,’ and orange depicts residues outside of domains. The ‘other sites’ group is comprised of sites depicted by blue and orange. Only orthogroups that contain at least one InteracDome domain were analyzed. **(C)** Orthogroups were filtered and aligned to build a final set of high confidence alignments (see Materials and methods), from which per-site evolutionary rates and variation were computed. To assess whether sites binding different types of ligands are under varying evolutionary pressure, evolutionary rates and variation of sites of different ligand-binding types were compared. **(D)** Human-specific variation—consisting of sites where a single amino acid is found across all primates (green circle) and an alternate amino acid is found in human (green star)—within ligand-binding sites was identified. **(E)** To assess whether human genes that exhibit human-specific variation in their ligand-binding sites tend to be associated with specific biological processes, functional enrichments were computed for these genes as compared to all other human genes in our ligand-binding dataset; we repeated this process for all sequences containing variable ligand-binding interfaces. Created with BioRender.com.

While these previous analyses have focused on sites involved in various protein-protein and specific protein-DNA interactions, the lack of site-wise ligand interface data for other types of protein interactions has prevented a broader global characterization of these interfaces. However, recent advances based on protein structural domains enable the inference of many ligand-binding sites within proteins. In particular, the InteracDome database aggregated protein-ligand interfaces by domain type and identified DNA-, RNA-, small molecule-, ion-, and peptide-binding positions within 4128 protein domains [58,59,60], of which 2152 are found in human protein sequences. We leverage the InteracDome to construct a comprehensive database of ligand-binding sites within primate single-copy proteins by transferring ligand-binding information within domains to a set of 4197 one-to-one primate orthologous sequence groups, creating a novel structurally-derived dataset that enables a large-scale evolutionary analysis of protein-ligand interfaces across primates using comparative genomic methods. Here, we use this new dataset to broadly characterize sites within the proteins of 18 primate species (Fig 1A) that interact with five critical ligand types: DNA, RNA, small molecules, ions, and peptides. Our work represents the largest characterization of the variation in protein-ligand interfaces to date.

Overall, we observe that the evolutionary rates across primates of sites that comprise protein-ligand interfaces are significantly slower than those of other sites and that interaction sites are significantly more likely to be completely conserved. Nevertheless, ~10% of interaction sites vary across primates, suggesting that interaction networks have evolved substantially across these species. Among the different types of ligand-binding sites studied, DNA and RNA interaction sites exhibit the slowest evolutionary rates. Nevertheless, we observe that proteins containing ligand-binding sites that are variable are enriched in several transcriptional regulatory functions. We further observe that proteins containing ligand-binding sites with amino acids that are human-specific or great ape-specific are also enriched in transcriptional regulatory proteins. These findings are consistent with previous hypotheses that changes in regulatory networks are a contributing factor to phenotypic variation in primates, while newly implicating changes in regulatory proteins themselves.

## Results

### Overview

In this section, we briefly describe our pipeline for the construction of a large, novel dataset of ligand-binding domain sites in one-to-one primate orthologs, along with how we used this dataset to systematically evaluate the evolutionary landscape of protein-ligand interfaces; see Materials and methods for details. First, we gathered all orthogroups consisting of at most one protein from each of 18 primate species (Fig 1A) from OrthoDB [61]. Second, we identified positions within these proteins that participate in interactions (Fig 1B). To do this, we used the InteracDome database [58], which aggregated co-complex structures by domain type, and identified positions within domains that interact with DNA, RNA, peptides, small molecules, or ions. Specifically, we identified these domains within each protein sequence, and transferred per-domain-position annotations of ligand binding to all residues that are mapped to these positions and annotated all other residues as background sites. Only orthogroups that contain at least one InteracDome domain were used analyzed. This identified 67,823 sites within these domains that bind ligands (denoted as ‘ligand-binding sites’), and identified 2,588,980 sites in all of the included orthogroups annotated as not binding ligands (denoted as ‘other sites’), of which 1,082,970 sites are located within the domains considered in this analysis (denoted as ‘other sites within domains’). Third, we aligned each orthogroup, and then for each site within each multiple sequence alignment (MSA), we quantified the amount of amino acid variation using Shannon entropy [62], and calculated maximum likelihood phylogenetic estimates of evolutionary rates [63] (Fig 1C). Fourth, we compared the amino acid variation and evolutionary rates of ligand-binding sites and other sites within domains, and compared these values across different ligand types, to characterize evolutionary pressures acting on these sites. We identified variable ligand-binding sites with amino acids that are unique to humans (Fig 1D). Finally, we identified orthogroups that contain variable ligand-binding sites and uncovered the biological processes in which they, and human-specific sites, are enriched (Fig 1E).

### Protein-ligand interfaces exhibit constrained evolution

In order to understand the evolutionary history of protein-ligand interfaces, we quantified the evolution of these interaction sites in the primary sequence space across 18 species of primates. We observed that ligand-binding sites are fully conserved at a significantly higher rate than other sites (89.2% vs. 77.3%, P < 0.001; Fisher’s exact test). However, our annotations of ligand-binding sites are based on domains, and domains are functional regions that may themselves be under evolutionary constraints; indeed, sites within domains tend to be more conserved than other sites (84.6% vs 77.3%, P < 0.001; Fisher’s exact test). Thus, throughout the remainder of our analysis, unless otherwise noted, we instead compare the evolutionary properties of ligand-binding sites to other sites within domains. All sites within the ‘other sites within domains’ group are located within the 2152 InteracDome domains we analyzed within human sequences; these domains have been observed in multiple co-complex crystal structures, and these sites have never been observed to bind ligands across these crystal structures. We find that ligand-binding sites are more likely to be fully conserved than other sites within domains (89.2% vs 84.6%, P<0.001; Fisher’s exact test). Despite the larger fraction of ligand-binding sites that are completely conserved, we find that 8.8%, 6.6%, 11%, 12% and 11% of sites that bind DNA, RNA, small molecule, ion and peptide, respectively, exhibit variation across the primates (Table 1); this suggests that there is potential for substantial variation in protein-ligand interactions across primates.

**Table 1:** Number of ligand-binding sites, other sites, and other sites within domains, and the fraction of these that are conserved. Data for ligand-binding sites are given both when considering all types of ligands together as well as when split up by type of ligand. Note that some sites may participate in interactions with more than one ligand type.

	Other Sites	Other Sites within Domains	Ligand-binding	DNA	RNA	Small Molecule	Ion	Peptide
**# of sites**	2,588,980	1,082,970	67,823	20,105	4,668	14,134	22,771	12,639
**% conserved**	77.26	84.63	89.23	91.22	93.38	88.96	87.95	88.96

Next we computed the maximum likelihood estimate of the evolutionary rate of amino acid sites in these sequences using the Rate4Site algorithm [63]. Rate4Site considers the phylogeny of the sequences, and higher scores correspond to faster evolutionary rates and higher amino acid variability. We found that ligand-binding sites are evolving more slowly than other sites within domains (means of 0.84 vs 0.90, respectively, P<0.001; Mann Whitney-U test (MWU)). We confirmed this result with a Shannon entropy [62] analysis across the same site groupings (means of 0.046 vs 0.064 for ligand-binding and other sites within domains respectively, P<0.001; MWU); with Shannon entropy, a score of 0 corresponds to no variation and higher scores correspond to sites with increasing amino acid diversity. The evolutionary conservation we have identified at ligand interfaces with both maximum likelihood and entropy-based measures of conservation and variation indicates a clear signal of purifying selection acting at ligand-binding sites.

### Different protein-ligand interface types exhibit significantly different evolutionary rates

**Fig 2:**
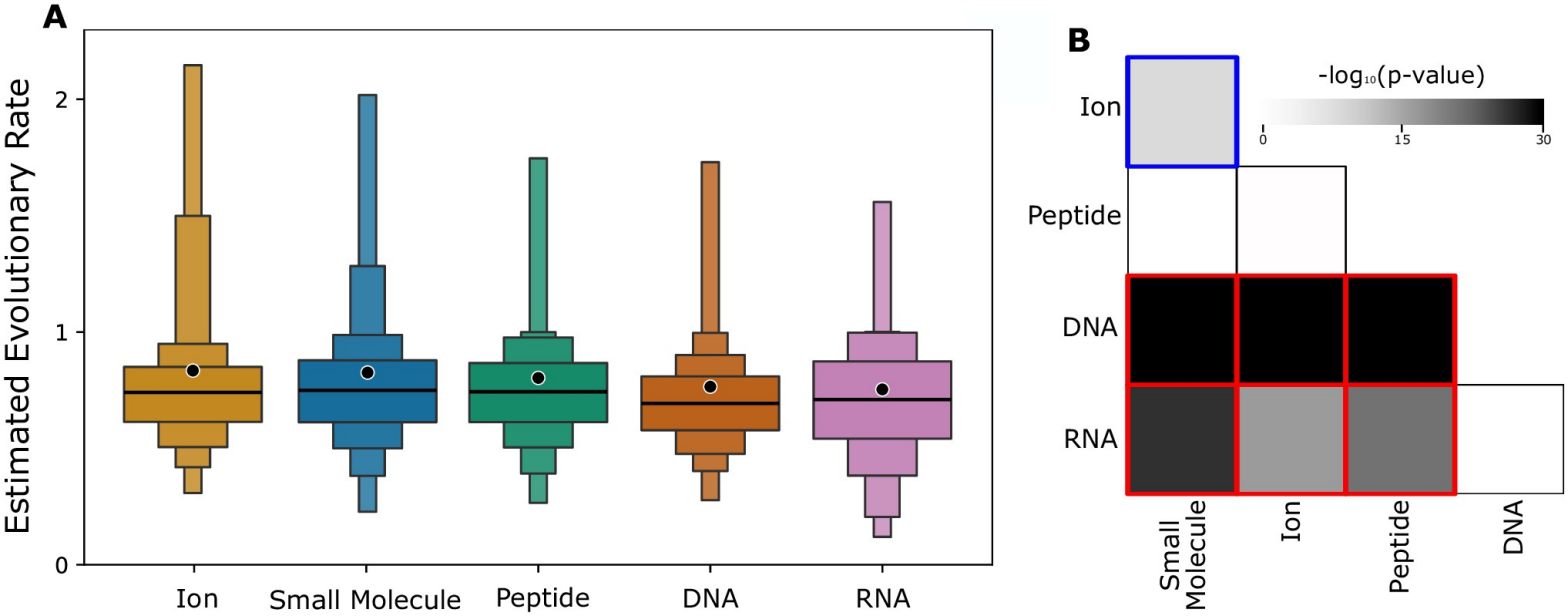
Relative evolutionary patterns of ligand-binding sites. **(A)** Letter-value (or boxen) plots of the estimated evolutionary rates computed by Rate4Site for sites that bind each of the five ligand types. In letter-value plots, the widest box shows half the data (from the 25th to 75th percentiles), while each successively narrower box shows half the remaining data. In the middle box for each ligand type, median values of estimated evolutionary rates are shown with a black line and mean values are shown with a black dot. Four letter-values (boxes) are plotted for visual clarity, in order of mean evolutionary rate (left to right). **(B)** Pairwise significance comparisons (Bonferroni corrected -log10(p-values), see ‘Materials and Methods’ section) of each ligand class are shown in greyscale, with outlined colored directional comparisons indicating if the ligand class on the bottom is more rapidly evolving than that on the left (red) or if the opposite is true (blue).

Having shown that as a group, the evolutionary rates of ligand-binding sites are lower than those for other sites within domains, we assessed the differences in evolutionary rates of different types of ligand-binding sites. There are notable differences in the evolutionary patterns between the classes of ligand-binding, with DNA- and RNA-binding sites showing the lowest evolutionary rates relative to other ligand types (Fig 2A). On average, sites involved in interactions with ions have the fastest evolutionary rates, followed by sites involved in binding small molecules and peptides. Pairwise comparisons show that DNA- and RNA-binding sites are evolving significantly more slowly than each of the three other reported ligand classes (Fig 2B).

### Distinct biological processes are associated with variable ligand-interfaces

To better understand the variation across ligand-binding sites, we identified 1160 orthogroups (28% of total) that contain at least one variable ligand-binding site, but this site does not exhibit common variation (minor allele frequency > 0.01) within the human population using the gnomAD database [66]; we reason that by excluding sites that have common variation across humans, we are more likely to exclude sites with neutral variation. Since these proteins contain ligand-binding interfaces that vary across primate species, we hypothesized that they may participate in broader functions that play a role in primate phenotypic variation. We used the PANTHER suite of tools [67] to perform a gene ontology (GO) enrichment analysis [68], with our set of genes containing ligand-binding domains as a background, to uncover the functional groups in which these variable sites are enriched (FDR < 0.05). Even within our restricted background gene set, we still see 42 significant functions arise (Table 2). Most notable among these functional groups are various terms associated with distinct transcriptional regulation activity including cis-regulatory region sequence-specific DNA binding, DNA-binding transcription factor activity, and RNA polymerase II DNA binding activity, all of which may be capable of significant downstream pleiotropic effects on primate phenotypes. We also observe more broad enrichments related to transcription including nucleic acid binding, zinc ion binding (and its parent terms transition metal ion binding, metal ion binding, cation binding and ion binding), and transcription regulator activity.

**Table 2:** GO terms for 1160 orthogroups with variable ligand interfaces. Significant GO terms (FDR < 0.05) are listed for proteins that contain at least one variable ligand contacting site.

GO Term	FDR
**ion binding**	3.99E-36
**metal ion binding**	1.06E-30
**cation binding**	1.62E-29
**catalytic activity**	1.14E-10
**heterocyclic compound binding**	1.62E-08
**organic cyclic compound binding**	3.88E-08
**calcium ion binding**	8.39E-07
**transition metal ion binding**	1.39E-04
**nucleic acid binding**	2.72E-04
**zinc ion binding**	2.90E-04
**catalytic activity, acting on a protein**	1.14E-03
**small molecule binding**	1.80E-03
**nucleoside phosphate binding**	1.89E-03
**nucleotide binding**	2.03E-03
**anion binding**	2.46E-03
**binding**	5.38E-03
**sequence-specific DNA binding**	5.51E-03
**DNA binding**	5.66E-03
**cis-regulatory region sequence-specific DNA binding**	6.15E-03
**RNA polymerase II transcription regulatory region sequence-specific DNA binding**	7.06E-03
**RNA polymerase II cis-regulatory region sequence-specific DNA binding**	7.13E-03
**DNA-binding transcription factor activity**	1.07E-02
**double-stranded DNA binding**	1.20E-02
**transcription cis-regulatory region binding**	1.24E-02
**DNA-binding transcription factor activity, RNA polymerase II-specific**	1.31E-02
**ATP binding**	1.36E-02
**carbohydrate derivative binding**	1.39E-02
**transcription regulatory region nucleic acid binding**	1.41E-02
**sequence-specific double-stranded DNA binding**	1.42E-02
**transcription regulator activity**	1.99E-02
**adenyl ribonucleotide binding**	2.31E-02
**adenyl nucleotide binding**	3.21E-02
**oxidoreductase activity**	3.24E-02
**catalytic activity, acting on a nucleic acid**	3.26E-02
**purine ribonucleoside triphosphate binding**	3.26E-02
**hydrolase activity**	3.29E-02
**catalytic activity, acting on a tRNA**	3.38E-02
**aminoacyl-tRNA ligase activity**	4.56E-02
**ligase activity, forming carbon-oxygen bonds**	4.66E-02
**integrin binding**	4.67E-02

Next, within the set of orthogroups with variable interaction sites, we divided the fraction of ligand-binding sites that are variable by the fraction of other sites within domains that are variable to calculate the relative fold enrichment of variable ligand-binding sites. This identified 73 orthogroups where variable sites were at least two-fold enriched in ligand-binding sites. Within this subset of orthogroups with an enrichment of variable ligand-binding sites, while there are no enriched functions, we see gene annotations involving signal transduction pathways and gene regulatory pathways (S1 Table).

### Ligand sites fixed in humans and great apes are involved in genetic regulatory mechanisms

To further understand how the evolutionary patterns of ligand contacts have contributed to the evolution of human-specific traits, we scanned all ligand-binding sites in all orthogroups for instances where there was a residue that was unique to humans and with an alternate amino acid fixed across the rest of the primate phylogeny. We then removed sites whose minor allele frequencies were > 0.01 (i.e., for which there are common variants) within the human population using the gnomAD database [66]. This approach identified 38 ligand-binding sites in 25 genes that were unique to humans at the evolutionary level as well as fixed within the human population. DNA, RNA, ion, small molecule, and peptide sites comprise 7.5%, 15%, 30%, 17.5% and 30% of this set of ligand contacts, respectively. Of these potential drivers of human-specific traits, we observed that six zinc finger transcription factors (ZFAT, ZNF638, ZFHX4, ZNF451, ZNF18, PEG3) comprise the largest individual gene group (S2 Table). Indeed, this set of 25 genes is enriched for transcription regulator activity within the background set of ligand-binding site containing genes (P < 0.05). Zinc fingers are thought to have an outsized contribution, even within regulatory pathways, to divergence in primate evolution [45]. While many previous studies focus on the gains and losses of transcription factors between humans and other primate species to identify regulatory network components responsible for divergence [44,45,46], our results present evidence for evolutionary drivers of human specific regulatory changes existing at ligand-binding sites even within genes that exhibit no gain or loss throughout the primate phylogeny. In particular, the observed set of six zinc fingers with human-specific changes indicates multiple methods of altering these genes’ functions through evolutionary changes at ligand interfaces. Three of these changes are in DNA-binding sites, suggesting that these particular changes may alter how the protein is interacting with a recognition sequence, while another one of these changes is an RNA-binding site in ZNF638, which has been experimentally shown to be directly involved in alternative splicing [69].

We performed a similar procedure on the ape clade to capture changes that may have driven clade-level divergence. Despite the number of fixed ligand-binding sites discovered in humans, no ligand-binding sites with one residue unique to the great apes and fixed across the rest of the primate phylogeny were found. We then relaxed these parameters to still require a unique amino acid to be fixed in the great apes, but the residues at the same site in the remainder of the primates could be any number of amino acids that do not overlap with the fixed great ape residue. This identified 14 ligand-binding sites in 13 genes (S3 Table). Similar to humans, we observe three zinc finger proteins (ZNF145, ZNF438, ZNF791), comprising the largest single gene type within the results, suggesting a possible change to transcriptional regulation mediated by each of these genes.

## Discussion

Protein-ligand interfaces are the site of many vital catalytic, enzymatic, and regulatory functions that are required for organismal function and likely have influence on evolutionary events throughout the primate phylogeny. Here we have shown that, overall, ligand-binding sites are significantly more conserved than other non-binding sites, and that the level of evolutionary constraint is dependent on the type of ligand the site binds, with DNA- and RNA-binding sites exhibiting the highest level of conservation. This suggests that DNA and RNA interfaces are under strong evolutionary constraints due to their large downstream impacts on transcriptional and translational networks. Despite this, approximately 9% of DNA- and 7% of RNA-binding sites have some variation across primates. Additionally, we have found that ligand-binding interfaces with amino acid variation are enriched in proteins involved in gene regulatory networks, suggesting that the variation in these interfaces may play a role in driving phenotypic differences between primate species.

Many DNA- and RNA-binding sites within proteins are constrained by recognition sequences or other biochemical properties, as changes within them could result in the gain or loss of numerous regulatory targets, and this is consistent with the purifying selection suggested by our results [70,71]. Ion contacts are often part of coordination complexes that also have biochemical constraints, but these contacts are likely more permissive to conservative amino acid substitutions that have similar charge. Further, peptide and small molecule interfaces may be more flexible to changes relative to nucleic acids, as DNA- and RNA-binding proteins can have thousands of targets [42,55] and therefore changes within sites participating in their interactions can result in pleiotropic effects. While there are still varying levels of steric constraints in peptide binding pockets and interfaces with small molecules and other peptides, there may be a lower level of baseline constraint acting on these two ligand interfaces compared to what is observed at DNA and RNA interfaces; peptide interfaces in particular tend to be larger with a few critical “hotspot” sites [72].

In addition to the biochemical constraints acting at ligand-binding interfaces, their participation in biological networks and the associated evolutionary constraints can also explain the patterns of conservation that we have observed. It has long been proposed that significant changes in protein interaction networks have taken place within primates and these changes likely play an important role in species evolution [18,73]. In particular, DNA and RNA interfaces are part of transcriptional and translational genetic regulatory pathways, suggesting that they are extremely sensitive to perturbations. Our results demonstrating the heightened conservation of DNA and RNA interfaces indicate that fewer of these interfaces vary within proteins relative to other ligand types, but is suggestive that in the cases that they are variable, they may be a contributing factor to phenotypic differences in primates through changes in gene regulatory pathways. It is possible that variation observed within these regulatory proteins could be due to weaker negative selection acting on them; this could arise, for example, if changes within transcription factors result in differing gene expression levels of downstream targets but these changes are mitigated by translational buffering [74]. However, because transcription factors can have hundreds or even thousands of gene targets [75], and mutations within transcription factors may also result in the gain of additional downstream targets, translational buffering is unlikely to fully compensate for the effects of variation within transcription factors.

While our characterization of the conservation of ligand binding sites indicates the sensitivity of ligand interfaces to evolutionary changes, it is the small percent of these sites that vary that are more informative for understanding the contribution of ligand interfaces to phenotypic differences in primates. Our analysis has shown that a subset of these variable interfaces are significantly enriched in a number of transcriptional regulatory proteins (Table 2) which is consistent with previous hypotheses that gene regulatory pathways contribute to phenotypic differences across primates, though here we are focused on changes within the regulators as opposed to the DNA or RNA being bound. The subset of these proteins containing variable ligand sites that we have identified with a two-fold or greater enrichment of variation in their ligand binding interfaces may be particularly interesting examples of sites with functionally relevant variation (S1 Table).

While DNA- and RNA-binding sites exhibit a broad signal of evolutionary constraint across primates, the enrichment of gene regulatory proteins in our human-specific gene set, particularly zinc fingers, provides evidence that even within this overall signal of conservation, these binding sites are showing species-specific evolutionary patterns in transcription factors. Similar to the outsized downstream effects observed from the existing body of work on changes in non-coding regulatory regions, the set of gene regulatory proteins in which we observe these fixed ligand-binding residues suggests that this small number of coding-region changes may also have a stronger downstream effect due to their action in gene regulatory pathways and thus have a larger resulting impact on the evolution of human-specific phenotypes.

While our analysis characterizes the evolutionary constraints acting on ligand contacting sites in one-to-one orthologs, the natural continuation of this work is to further describe the evolutionary landscape of protein-ligand interfaces across duplication and loss events in multi-copy orthologs containing ligand-binding interfaces within the primate phylogeny. It is possible that using multi-copy orthologs would strengthen our inferential power past individual speciation events, and provide a deeper understanding of how duplications and losses of ligand-binding interaction networks influence the phenotypic evolution of primate species. Finally, the study of multi-copy orthologs would allow for the expansion of this analysis to groups of more distantly related organisms—as the corresponding orthogroups typically contain multiple sequences for some species—and would help unravel the contribution of variation within protein sequences to network and phenotypic variation across large evolutionary distances.

## Materials and methods

### Annotation of orthogroups

To build a sequence space across a set of species to characterize the evolutionary landscape of ligand-binding sites, we first retrieved all one-to-one orthologous gene groups across 18 primate species (including human) (Fig 1A) from OrthoDB, a curated hierarchical catalog of orthologs [61]. This yielded an initial set of 5786 orthogroups that contain at least one domain for which binding positions have been annotated by InteracDome [58]. Ligand-binding sites within these orthogroups are annotated as described next, with the set of orthogroups filtered so as to maintain high quality annotations.

### Dataset of ligand-binding positions in orthogroups

Ligand-binding domains in human sequences are identified as follows. Domains in the primary sequence space were identified using HMMER [58,76]. Within the InteracDome [58], for each domain-ligand pair, each domain position is assigned a per-position binding frequency based on the distances of all residues in that position to the appropriate ligand across all available co-complex structures; these per-position binding frequencies correspond to the fraction of co-complex crystal structures where the amino acid at that position is in contact with the ligand. For each corresponding domain position in our analysis that is also annotated by the InteracDome, we identified DNA, RNA, peptide, and small molecule-binding positions using a binding frequency cutoff of at least 10% that also resulted in a 0.5 cross-validation precision threshold as determined by InteracDome. For ion-binding sites, we used the same frequency cutoff but a cross-validation threshold corresponding to 0.75 precision to increase the certainty of these sites as true ligand-binding sites (a higher threshold is used for ion-binding sites to reduce the number of false positives due to ion coordination distances in co-complex structures) [77]. Positions within domains were labeled as non-binding if the contact frequency is 0; all positions with above zero frequency but below the cross-validation threshold are excluded from the analysis. Note that every domain position that is labeled as contacting a particular ligand is observed to do so in co-complex crystal structures, and every domain position that is labeled as not contacting a ligand has never been observed to do so in any co-complex crystal structure. Using these annotations of positions within domains, domain-level ligand-binding and non-binding information was transferred to each primary human amino acid sequence in our dataset.

We increased per-site confidence of transferring human ligand-contacts in each orthogroup through a series of alignment and filtering steps. We built an MSA of each orthogroup with ClustalW using default parameters [78]. To improve these alignments at the sequence level, we performed a global pairwise alignment of each primate species to the human sequence in each orthogroup using the EMBOSS Needle program [79]. Sequences that did not pass a threshold of 80% similarity (sites that are either identical or biochemically conservative substitutions) or have more than 10% gaps in the alignment were removed from the orthogroup. Next, for every orthogroup, each position in each domain in all primate species was checked to ensure that the match state of each primate was aligned to the corresponding match state in the human sequence of that orthogroup. Sequences that were shifted during alignment such that the domain match states did not align were removed from the orthogroup. All orthogroups with fewer than 10 remaining sequences were excluded from the analysis. With this filtering procedure, we built a confident final data set of 4197 orthogroups on which our global analysis was conducted. These orthogroups contained 67,823 ligand-binding sites (i.e., this many columns in the MSAs correspond to ligand-binding sites). While our primary analysis focuses on these ligand-binding sites and compares them to other non-binding sites within domains (denoted as other sites within domains), we also consider the set of sites which includes both non-binding sites within domains as well as all sites within these orthogroups that are not within InteracDome domains (denoted as other sites).

### Quantifying the evolutionary rate of ligand-contacts

We extracted our 18 primate species subtree from a putative primate phylogeny of 186 species [64] using the ete3 phylogenetics package (Fig 1A) [80]. For all columns in each aligned orthogroup, we calculated a maximum likelihood estimate of the per-site evolutionary rate of each residue using the Rate4Site algorithm [63]. Human is set as the reference sequence and the branch lengths of the species tree are fixed to those obtained from the reference primate phylogeny. Using the same alignments, we also calculated the per-site Shannon entropy with no sliding window and no gap penalties or cutoffs [62].

Per-site output was aggregated into a classification of ligand-binding sites, other sites, and other sites within domains, which were the basis of our analysis, as follows. We first grouped all columns in ligand-binding sites, other sites and other sites within domains in the MSA into sets of sites with and without amino acid variation and tested for global differences in variability between ligand-binding sites and the other two groups of sites using a Fisher’s exact test. We then tested for significant differences in the maximum likelihood evolutionary rates of ligand-binding sites and other sites within domains by aggregating all sites into these two classifications and performing a Mann-Whitney U test. To test for differences in the evolutionary rate of different classes of ligand contacts, we used a Kruskal-Wallis test on the matrix of all ligand types to identify whether there was a significant difference between any ligand type. We followed this with an all-against-all post-hoc Conover test of multiple comparisons with a Bonferroni correction to identify which individual ligand type evolutionary rate comparisons were significant.

### Identifying instances of both evolutionarily fixed and variable binding sites

We identified sites that have human-specific conservation patterns. That is, these are sites that have a distinct amino acid in human and a divergent, but fixed, amino acid across all other primate species. We parsed all ligand-binding sites in every one of the total 4197 orthogroups for these sites that are unique to humans. To then identify sites that are fixed in the human population, we identified all sites that have single nucleotide polymorphisms in the gnomAD database [66] with an allele frequency ≤ 0.01 and are not annotated as disease-associated SNPs in gnomAD; by removing sites with human-specific variation from these fixed sites, we built a dataset that is comprised of sites that are fixed at both the non-human primate evolutionary level and the human population level. We further identified sites that are fixed at the clade-level within the great apes, similarly meaning a residue that is fixed as a distinct amino acid within the great apes, and a separate distinct amino acid across the rest of the primate phylogeny. We then removed the latter constraint of a fixed distinct amino acid across the remainder of the primate phylogeny in a follow-up analysis.

We also identified proteins with variable ligand-binding sites. That is, these are proteins with ligand-binding sites for which there is at least one amino acid change within the 18 primate species phylogeny. Biologically active sites that are variable may be candidates for sites that are responsible for phenotypic differences between species. We next used the PANTHER suite of gene ontology tools to identify significantly enriched gene ontology terms in the resulting set of genes (defined as those with false discovery rate or FDR < 0.05) [81,67], using the human proteins in our conserved set of orthogroups as the background (i.e., those orthogroups that are included in our broader analysis). All reported *p* values and FDRs for functional enrichment are computed by PANTHER. Finally, we calculated the fold-enrichment of variable ligand-binding sites relative to variable non-binding sites, and identified instances where a protein contained multiple variable ligand-binding sites and is at least two-fold enriched in ligand-binding sites.

## Supporting information

S1 Fig Histogram of the number of sequences in each multiple sequence alignment comprising the total set of 4197 orthogroups after filtering. Filtering sequences increases confidence in the quality of the alignments and the information at each site but reduces orthogroup size. The most frequent orthogroup size after filtering is 15 of the original 18 sequences.
(PDF)

S1 Table 73 proteins containing multiple ligand interfaces with at least two-fold enrichment of these variable sites. Gene IDs and descriptions are shown for proteins with ligand-binding sites at least two-fold enriched for amino acid variation vs non-binding sites in the same protein and with multiple variable sites.
(XLSX)

S2 Table Genes containing ligand-binding sites that differ from the residue that is conserved across the other primates but that are also fixed across human populations. There are a total of 38 sites within 25 genes that have human-specific amino acids that are also fixed across the human population; the ID, name, and description of each gene containing at least one fixed ligand-binding site are listed. The type of ligand contacting site such that it has an amino acid that is unique in humans is also provided, as well as the numbers of each site that are variable, which are shown in the corresponding order. There are three instances of a single site contacting two different ligands.
(XLSX)

S3 Table Genes containing ligand-binding sites conserved across the great apes and unconstrained across the rest of the primate phylogeny. There are a total of 14 sites fixed within 13 genes; the ID, name, and description of each gene containing at least one fix ligand-binding site are listed.
(XLSX)
