# Supplementary figures and images for: Primate protein-ligand interfaces exhibit significant conservation and unveil human-specific evolutionary drivers
Source: PLoS Comput Biol. 2023 Mar 23;19(3):e1010966. doi: 10.1371/journal.pcbi.1010966 (PMC10035887; doi:10.1371/journal.pcbi.1010966)

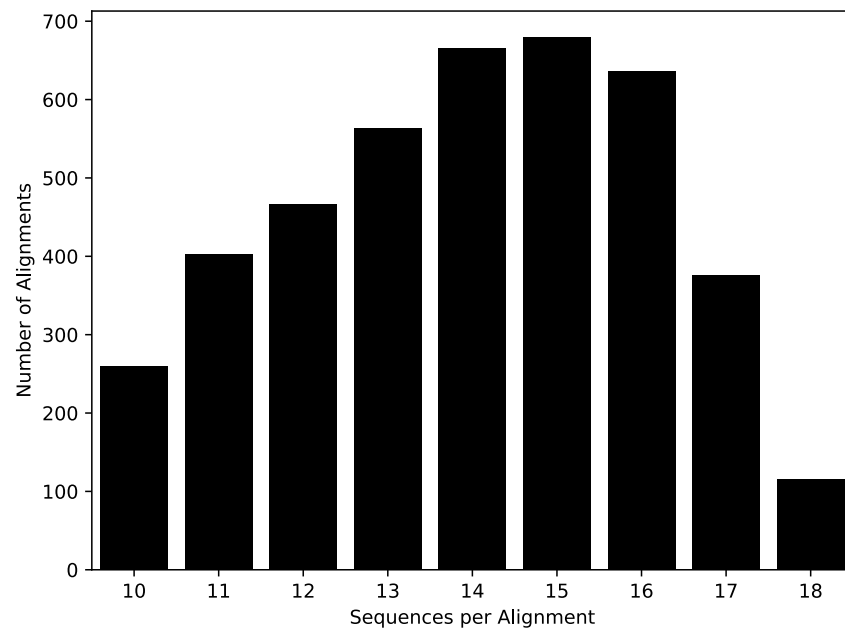

Supplement: S1 Fig — Filtering sequences increases confidence in the quality of the alignments and the information at each site but reduces orthogroup size. The most frequent orthogroup size after filtering is 15 of the original 18 sequences. (PDF) [file pcbi.1010966.s001.pdf]
